# Siccanin Is a Dual-Target Inhibitor of *Plasmodium falciparum* Mitochondrial Complex II and Complex III

**DOI:** 10.3390/ph15070903

**Published:** 2022-07-21

**Authors:** Keisuke Komatsuya, Takaya Sakura, Kazuro Shiomi, Satoshi Ōmura, Kenji Hikosaka, Tomoyoshi Nozaki, Kiyoshi Kita, Daniel Ken Inaoka

**Affiliations:** 1 Department of Biomedical Chemistry, Graduate School of Medicine, The University of Tokyo, Tokyo 113-0033, Japan; komatsuya-ks@igakuken.or.jp (K.K.); nozaki@m.u-tokyo.ac.jp (T.N.); 2 Laboratory of Biomembrane, Tokyo Metropolitan Institute of Medical Science, Tokyo 156-8506, Japan; 3 Department of Molecular Infection Dynamics, Institute of Tropical Medicine (NEKKEN), Nagasaki University, Nagasaki 852-8523, Japan; takaya.sakura@nagasaki-u.ac.jp; 4 School of Tropical Medicine and Global Health, Nagasaki University, Sakamoto, Nagasaki 852-8523, Japan; 5 Graduate School of Infection Control Sciences, Kitasato University, Tokyo 108-8641, Japan; shiomi@bikaken.or.jp; 6 Ōmura Satoshi Memorial Institute, Kitasato University, Minato-ku, Tokyo 108-8641, Japan; omuras@insti.kitasato-u.ac.jp; 7 Department of Infection and Host Defense, Graduate School of Medicine, Chiba University, Chiba 260-8670, Japan; hikosaka@chiba-u.jp; 8 Department of Host-Defense Biochemistry, Institute of Tropical Medicine (NEKKEN), Nagasaki University, Nagasaki 852-8523, Japan

**Keywords:** malaria, mitochondria, electron transport chain, complex II, complex III, drug target

## Abstract

*Plasmodium falciparum* contains several mitochondrial electron transport chain (ETC) dehydrogenases shuttling electrons from the respective substrates to the ubiquinone pool, from which electrons are consecutively transferred to complex III, complex IV, and finally to the molecular oxygen. The antimalarial drug atovaquone inhibits complex III and validates this parasite’s ETC as an attractive target for chemotherapy. Among the ETC dehydrogenases from *P. falciparum*, dihydroorotate dehydrogenase, an essential enzyme used in de novo pyrimidine biosynthesis, and complex III are the two enzymes that have been characterized and validated as drug targets in the blood-stage parasite, while complex II has been shown to be essential for parasite survival in the mosquito stage; therefore, these enzymes and complex II are considered candidate drug targets for blocking parasite transmission. In this study, we identified siccanin as the first (to our knowledge) nanomolar inhibitor of the *P. falciparum* complex II. Moreover, we demonstrated that siccanin also inhibits complex III in the low-micromolar range. Siccanin did not inhibit the corresponding complexes from mammalian mitochondria even at high concentrations. Siccanin inhibited the growth of *P. falciparum* with IC_50_ of 8.4 μM. However, the growth inhibition of the *P. falciparum* blood stage did not correlate with ETC inhibition, as demonstrated by lack of resistance to siccanin in the yDHODH-3D7 (EC_50_ = 10.26 μM) and Dd2-ELQ300 strains (EC_50_ = 18.70 μM), suggesting a third mechanism of action that is unrelated to mitochondrial ETC inhibition. Hence, siccanin has at least a dual mechanism of action, being the first potent and selective inhibitor of *P. falciparum* complexes II and III over mammalian enzymes and so is a potential candidate for the development of a new class of antimalarial drugs.

## 1. Introduction

Human falciparum malaria, caused by *Plasmodium falciparum*, accounts for an estimated 241 million cases and 627,000 deaths annually. Most of these cases are children under the age of five in developing countries [1]. Unfortunately, the number of cases and deaths is presumed to have increased during the COVID-19 pandemic as a result of disruptions in the provision of malaria prevention, diagnosis, and treatment. Currently, the World Health Organization (WHO) recommends that malaria be treated with a combination therapy consisting of artemisinin and other antimalarial drugs (Artemisinin Combination Therapy; ACT). Moreover, WHO’s goal of malaria control appears to be far from being achieved because no effective vaccine is yet available, and *P. falciparum*, with artemisinin resistance, is emerging in southeastern Asia [2] and has recently independently emerged in Africa [3,4,5]. Hence, new drugs with different or multiple mechanisms of action are needed to control malaria. In that sense, drug targets with distinct biochemical features or even those that are absent from the host are desired. Currently, all approved antimalarial drugs were developed targeting the *P. falciparum* blood stage with some, such as atovaquone, proguanil (prodrug of cycloguanil), pyrimethamine and doxycycline, found to be very effective prophylactic agents killing liver schizonts. However, for the elimination of malaria, novel interventions targeting several stages (e. g., the mosquito and liver stages) as well as the blood stage of *P. falciparum* are required [6].

**Figure 1:**
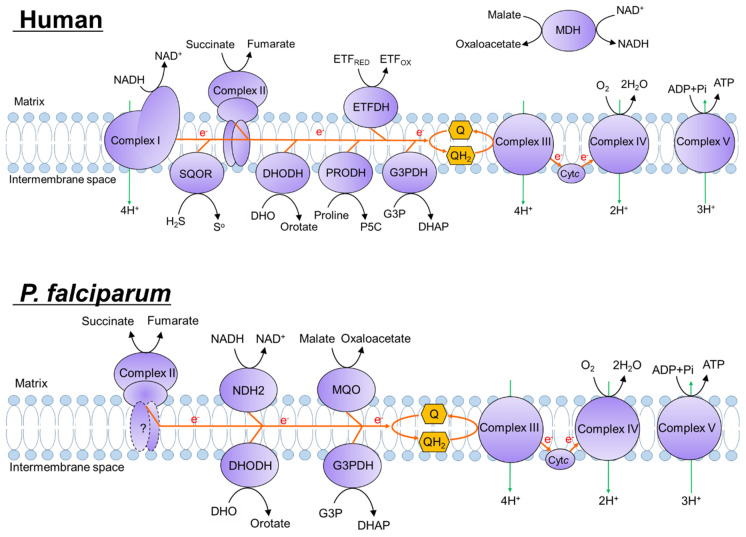
Schematic representation of the mitochondrial electron transport chain in human (**top**) and *P. falciparum* (**bottom**). Orange and green arrows indicate the flow of electrons and protons, respectively. The anchor subunits of complex II (CybL and CybS) from *P. falciparum* have yet to be identified. The reactions mediated by complex II are known to be reversible, such that complex II can act as a succinate:quinone reductase (SQR, forward reaction) or a quinol:fumarate reductase (QFR, reverse reaction). Genes encoding the plasmodial homologues of human SQOR, MDH, PRODH, ETF, and complex I are missing from the *P. falciparum* genome, as are the genes encoding human homologues of *P. falciparum* NDH2, and MQO from the human genome. NADH, reduced nicotinamide adenine dinucleotide; NAD^+^, oxidized nicotinamide adenine dinucleotide; DHO, dihydroorotate; DHODH, DHO dehydrogenase; P5C, (S)-1-pyrroline-5-carboxylate; PRODH; proline dehydrogenase; SQOR, sulfide:quinone oxidoreductase; G3P, glycerol-3-phosphate; DHAP, dihydroxyacetone phosphate; G3PDH, G3P dehydrogenase; ETF, electron transfer flavoprotein; ETFDH, ETF dehydrogenase; Q, ubiquinone; QH_2_, ubiquinol; Cyt *c*, cytochrome c; MDH, soluble malate dehydrogenase; MQO, malate:quinone oxidoreductase; NDH2, type-II NADH dehydrogenase; ADP, adenosine diphosphate; Pi, inorganic phosphate; and ATP, adenosine triphosphate.

The electron transport chain (ETC) of the parasite’s mitochondrion is highly diverse. In *P. falciparum*, the ETC is composed of five dehydrogenases, including type-II NADH dehydrogenase (NDH-2) [7], succinate dehydrogenase (SDH or complex II) [8,9], malate:quinone oxidoreductase (MQO) [10], dihydroorotate dehydrogenase (DHODH) [11], and glycerol-3-phosphate dehydrogenase (G3PDH) [12]; collectively, these enzymes shuttle electrons to the ubiquinone pool (Q-pool) (Figure 1). Among plasmodial ETC dehydrogenases, NDH2 and MQO are not present in the mammalian cell [13], and SDH [14] and DHODH display biochemical features distinct from the mammalian homologues and do not share cross-sensitivity to inhibitors with the mammalian enzymes (Figure 1) [15,16,17,18,19,20]. Little is known about the role of the G3PDH in the plasmodial ETC; however, it has been suggested that this enzyme plays a role in lipid biosynthesis and redox homeostasis in the parasite [12]. The antimalarial drug atovaquone inhibits mitochondrial complex III (cytochrome *bc*_1_ complex or quinol: cytochrome *c* reductase), which transfers electrons from the Q-pool to cytochrome *c* [21]. We previously demonstrated that complex II is not essential for the survival of *P. falciparum* [9] and *P. berghei* [8] blood stages. However, it is important to note that a knockout in the gene encoding the Fp subunit of complex II (*sdha*) causes growth retardation in the blood stage; this defect is rescued by the addition of succinate but not by fumarate [9], suggesting that complex II may function as a quinol-fumarate reductase (QFR) rather than as a succinate-quinone reductase (SQR) [22]. Similarly, *sdha* knockout *P. berghei* shows a delayed onset in the initial parasitemia, which subsequently returns to wild-type levels within three days [8]. In the mosquito stage, *sdha* knockout *P. berghei* is not able to develop into oocysts within the vector [8], indicating that both a functional ETC and the tricarboxylic acid (TCA) cycle are essential for parasite development and survival in the insect stage. Thus, complex II is considered a novel target for development of transmission-blocking agents with activity against plasmodial infection. Consistent with this hypothesis, malaria parasites resistant to atovaquone have reduced complex III activity and failed to develop into oocysts, and, thus, are unable to spread the disease [21]. In addition, atovaquone potently inhibits the development of sporozoites into merozoites in the liver stage [23]. Thus, the ETC of *P. falciparum* includes enzymes that are essential at multiple stages and therefore may be considered a promising drug target for the development of new antimalarials.

Mitochondrial complex II participates in the TCA cycle, transferring electrons from succinate to ubiquinone, thereby producing fumarate and ubiquinol (Figure 1) [24,25]. Interestingly, the subunit composition of complex II is highly diverse. For instance, in mammals and nematodes, complex II consists of four subunits [25] (flavoprotein (Fp); iron-sulfur cluster protein (Ip); and two membrane anchor subunits, the cytochrome *b* large (CybL) and small (CybS) subunits), whereas in apicomplexan parasites *Toxoplasma gondii* and *Eimeria tenella*, complex II consists of 9 and 15 subunits, respectively [26,27,28]. A previous report suggested the presence of a four-subunit-type complex II in *P. yoelii yoelii* [14]. However, in *P. falciparum*, only the Fp and Ip subunits have been identified to date, but not the CybS and CybL subunits, indicating low sequence similarity among the CybL and CybS subunits, even among *Plasmodium* species. Previously, we identified atpenin A5 as the first potent and specific inhibitor of complex II from mammals and nematodes [29]. Surprisingly, atpenin A5 and other classical complex II inhibitors, such as 2-thenoyltrifluoroacetone thenoyltrifluoroacetone (TTFA) and carboxin, are not effective against complex II from *Plasmodium* [14,30] (Table 1) and *E. tenella* [27], indicating that the structure of the ubiquinone-binding site of complex II is significantly divergent between apicomplexan parasites and mammals.

**Figure 2:**
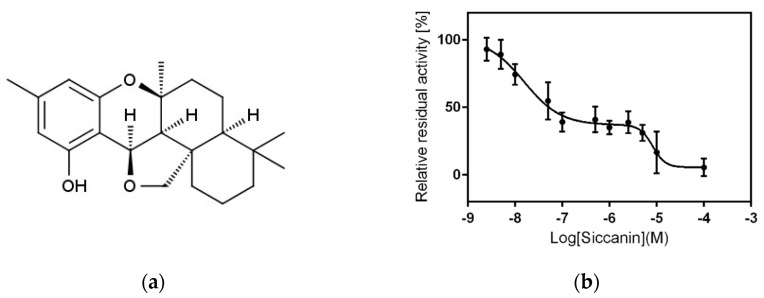
Siccanin is a potent inhibitor of *Plasmodium falciparum* complex II. (**a**) Chemical structure of siccanin. (**b**) Biphasic inhibition of succinate:ubiquinone reductase (SQR) activity by siccanin. Using a crude mitochondrial fraction isolated from parasite culture, the SQR activity was determined by monitoring 2,6-dichlorophenolindophenol (DCIP) reduction (see Section 4 for detailed information). The 50% inhibitory concentrations (IC_50_s) of 0.016 ± 0.006 μM and 8.93 ± 2.44 μM were calculated using the biphasic dose-response equation in GraphPad Prism^®^ ver.6.01. Data are presented as mean ± SD (*n* = 3).

The development of drugs targeting complex II has been reported for compounds with activity against many pathogens, including *Ascaris suum* [29], *Trichophyton mentagrophytes* [31], *Mycobacterium tuberculosis* [32], and *Helicobacter pylori* [33]. In the present study, we identified siccanin (Figure 2a) as the first (to our knowledge) nanomolar and selective inhibitor of the *P. falciparum* complex II. We also showed that siccanin inhibits complex III at micromolar concentrations. Moreover, we demonstrated that siccanin inhibits the growth of blood-stage *P. falciparum*, an effect that appears to be distinct from the ETC inhibitory activity. Therefore, siccanin is a promising lead compound for the development of new antimalarial drugs for the treatment of *Plasmodium*, including the potential for blocking parasite transmission.

**Table 1:** Selectivity of classical complex II inhibitor against *Plasmodium* spp. and mammal complex II.

Inhibitor	Complex II IC_50_ [μM]		Selectivity ^a^
	*Plasmodium* spp.	Mammal	
Malonate	13.2 ± 0.49 ^b^	3.4 ^d^	0.26
TTFA	>50 ^b^	5.4 ^e^	<0.11
Atpenin A5	4.6 ± 0.2 ^c^	0.004 ^e^	0.00087
Carboxin	3.6 ± 1.0 ^c^	1.0 ^e^	0.28

^a^ Selectivity was calculated as the ratio between mammalian cell line and *P. falciparum* 50% inhibitory concentrations (IC_50_s). Adapted from Suraveratum et al. [30] ^b^, Kawahara et al. [14] ^c^, Mogi et al. [34] ^d^, and Miyadera et al. [29] ^e^.

## 2. Results

### 2.1. Siccanin Strongly Inhibits P. falciparum SQR Activity

**Figure 3:**
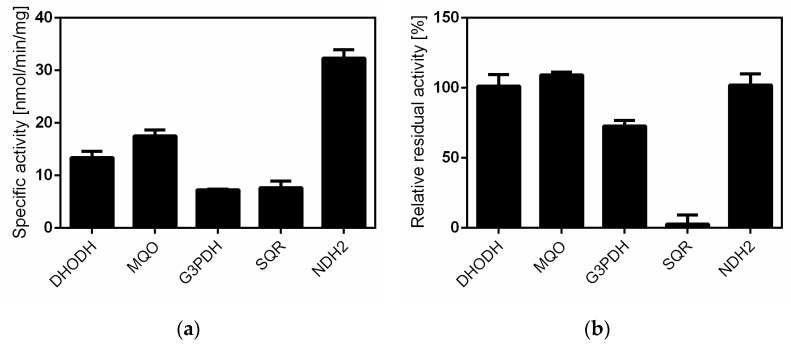
Siccanin is a specific inhibitor of *P. falciparum* complex II amongst *P. falciparum* electron transport chain (ETC) dehydrogenases. (**a**) Specific activities of five ETC dehydrogenases from a *P. falciparum* mitochondria-rich fraction. Specific activities of dihydroorotate dehydrogenase (DHODH), malate:quinone oxidoreductase (MQO), glycerol-3-phosphate dehydrogenase (G3PDH), and succinate:quinone oxidoreductase (SQR) were determined by monitoring the absorbance change associated with reduction of 2,6-dichlorophenolindophenol (DCIP). The type-II NADH dehydrogenase (NDH2) activity was determined by monitoring the reduction of NADH (see Section 4). DHODH-, MQO-, G3PDH-, SQR-, and NDH2-specific activities are 13.4 ± 1.2, 17.5 ± 1.1, 7.25 ± 0.1, 7.63 ± 1.3, and 32.3 ± 1.6 nmol/min/mg protein, respectively. (**b**) The inhibitory effect of siccanin at a concentration of 10 μM against the five dehydrogenases. Relative residual activities (%) of DHODH, MQO, G3PDH, SQR, and NDH2 were 101.2 ± 8.1, 109.2 ± 2.0, 72.5 ± 4.0, 2.7 ± 6.5, and 101.8 ± 8.1 nmol/min/mg protein, respectively. Data are presented as mean ± SD (*n* = 3).

Siccanin (Figure 2a), an antibiotic produced by the plant pathogenic fungus *Helminthosporium siccans* Dreschsler [35,36], was previously reported to be a potent and selective inhibitor of fungal, trypanosomal, and nematode complex II [37,38,39,40] and has been used clinically for the treatment of tinea pedis (Tackle^®^, Sankyo-Pharma). Our previous study showed that siccanin is a species-selective complex II inhibitor, effective against complex II from trypanosomatid parasites [38], *Pseudomonas aeruginosa*, *P. putida*, rat, and mouse but ineffective against the enzymes from *Escherichia coli*, *Corynebacterium glutamicum*, and porcine [34]. Therefore, we examined whether siccanin inhibits *P. falciparum* SQR activity in a crude mitochondrial fraction from *P. falciparum* 3D7. We found that siccanin strongly inhibited the SQR activity of this fraction (Figure 2b). In contrast, classical ubiquinone-site inhibitors, such as TTFA [30], atpenin and carboxin, were ineffective against the *Plasmodium* mitochondrial complex II [14] (Table 1). The inhibition by siccanin exhibited a classical biphasic inhibition pattern with 50% inhibitory concentration (IC_50_) values (mean ± SD) of the first and second phases of 0.016 ± 0.006 µM and 8.93 ± 2.44 µM, respectively (Figure 2b and Table 2). As a next step, the effect of siccanin at a concentration of 10 µM against other mitochondrial dehydrogenases (Figure 3a,b) was evaluated, revealing that siccanin did not inhibit MQO, DHODH, and NDH2 activities but weakly inhibited G3PDH (Figure 3b). These results clearly showed that siccanin is a selective inhibitor of the *P. falciparum* complex II amongst the mitochondrial ETC dehydrogenases that shuttle electrons to the Q-pool (Figure 3b). Our results also indicated that the *P. falciparum* complex II has higher sensitivity to siccanin than does the enzyme from *T. mentagrophytes* (IC_50_~90 nM) [31].

**Table 2:** Selectivity of siccanin against complexes II and III, and also the growth of *P. falciparum* 3D7 and mammalian cells.

Assay	Siccanin IC_50_ [μM]		Selectivity ^a^
	*P. falciparum*	Mammal	
Complex II	0.016 and 8.93	861 ^b^	57,400 and 96
Complex III	8.39	>500	>60
Growth	8.4	34.2 ^c^ 16.1 ^d^	4.1 1.9

^a^ Selectivity was calculated as the ratio between mammalian cell line and *P. falciparum* 50% inhibitory concentrations (IC_50_s). ^b^ Adapted from Mogi et al. [34]; ^c^ DLD-1 cells; ^d^ HDF cells.

### 2.2. Inhibition of P. falciparum Growth by Siccanin

**Figure 4:**
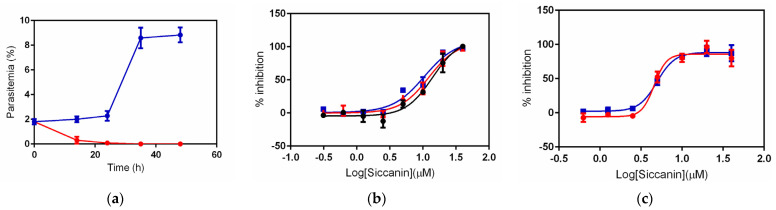
*P. falciparum* complex II is not the primary target of siccanin at the blood stage. (**a**) Synchronized parasites were incubated for 16, 24, 32, and 48 h with vehicle (dimethyl sulfoxide; DMSO) (blue) or 50 µM siccanin (red) and parasitemia was measured by Giemsa staining. (**b**) *P. falciparum* 3D7 was incubated for 72 h with different concentrations of siccanin in the absence (black) and presence of either 5 mM succinate (blue) or 5 mM fumarate (red). The IC_50_s were calculated as 8.40 ± 0.60, 10.8 ± 2.16, and 11.5 ± 1.09 μM, respectively. (**c**) Effect of siccanin on the growth of *Δsdha*-3D7 (red) and the parent (wild-type) 3D7 (blue). The IC_50_s were calculated as 6.13 ± 0.91 μM and 6.10 ± 1.00 μM for *Δsdha*-3D7 and wild-type parasites, respectively, using the four-parameter logistic equation in GraphPad Prism^®^ ver.6.01. Data are presented as mean ± SD (*n* = 3).

In other organisms living in anaerobic or microaerophilic environments (e.g., *E. coli* [41], *Mycobacterium tuberculosis* [32,42,43,44], *Ascaris suum* (adult stage) [29], *Echinococcus multilocularis* (protoscoleces) [45], and even in several solid tumor cells [46]), the QFR activity of complex II is well documented and has been suggested to play an important role in environmental adaptation. Previously, we demonstrated that the disruption of the Fp subunit-encoding gene of *P. falciparum* (*sdha*) impairs the growth of blood-stage parasites; the growth of the *Δsdha* mutant was rescued by succinate but not by fumarate, suggesting that this plasmodial complex II might function as a QFR rather than as a SQR [9]. Since siccanin potently inhibited complex II from *P. falciparum*, we next tested whether siccanin exposure phenocopied the *Δsdha* mutant. As described above, complex II is not essential for the survival of blood-stage *P. falciparum*; however, we could not detect live parasites under the microscope following exposure to siccanin at a concentration of 50 µM (Figure 4a). Further experiments demonstrated that siccanin modestly inhibits the growth of *P. falciparum* with an IC_50_ of 8.40 ± 0.60 µM (Figure 4b, Table 2). This result suggested that siccanin may have a secondary target other than complex II in *P. falciparum*; presumably this second target is essential for survival in the blood stage.

### 2.3. Effect of Succinate or Fumarate, and Pfsdha Disruption, on Growth Inhibition by Siccanin

Next, we tested whether addition of succinate or fumarate [9] rescues the growth-inhibitory effect of siccanin (Figure 4a). Compared to the control’s IC_50_ of 8.4 μM, the IC_50_ of siccanin in the presence of succinate or fumarate was 10.8 or 11.5 μM, respectively (Figure 4b), suggesting that the 3D7-growth inhibition caused by siccanin was independent of the compound’s effect on complex II activity. Consistent with this hypothesis, the effect of siccanin on *Δsdha*-3D7 *P. falciparum* growth was similar to that on the parent 3D7, with IC_50_s of 6.13 and 6.10 μM, respectively (Figure 4c). Together, these results strongly indicated the existence of a secondary essential target of siccanin in the blood-stage parasite.

### 2.4. Siccanin Inhibits P. falciparum Complex III

**Figure 5:**
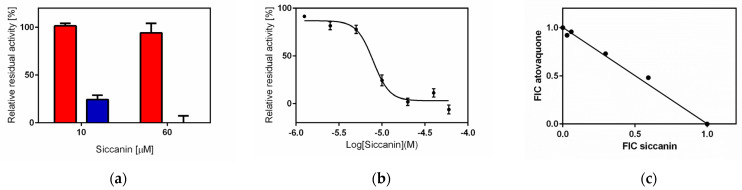
Inhibition of complex III by siccanin and its isobologram versus atovaquone. (**a**) Siccanin does not inhibit dihydroorotate dehydrogenase activity (red) but does exhibit dose-dependent inhibition of dihydroorotate–cytochrome *c* activity (corresponding to the coupled activity of DHODH and complex III) (blue). (**b**) The 50% inhibitory concentration (IC_50_) of siccanin against complex III was determined as 8.39 ± 2.92 μM using the four-parameter logistic method in GraphPad Prism^®^ ver.6.01. Data are presented as mean ± SD (*n* = 3). (**c**) Isobologram analysis showing the combinatory effect of atovaquone and siccanin against the *P. falciparum* 3D7 strain (*n* = 3). Normalized fractional inhibitory concentration (FIC) index values were calculated as described in Section 4. The line connecting the FIC = 1.0 points on the two axes represents the line of additivity. The plots of FIC calculated for siccanin in the presence of varying concentrations of atovaquone are shown as black circles, which coincide with the line of additivity. This result clearly indicates an additive effect when parasites are treated with a combination of siccanin and atovaquone.

DHODH, MQO, and complex III (Figure 1) are ETC enzymes that have been suggested to be essential for the survival of the blood-stage *P. falciparum* [11,47]. Since siccanin did not inhibit DHODH and MQO activities (Figure 3b), we tested whether complex III activity is inhibited by siccanin. The inhibition of complex III was evaluated using DHO-cytochrome *c* reductase. This assay measures electron transfer from DHODH to cytochrome *c* via complex III by recording the absorbance change of cytochrome *c* at 550 nm [14]. The results showed that, at 60 μM concentration, siccanin does not inhibit DHODH activity (Figure 5a) but completely inhibits DHO-cyt *c* activity with an IC_50_ value of 8.39 ± 2.92 μM (Figure 5b, Table 2), indicating that siccanin inhibits complex III and not DHODH. It has been reported that siccanin does not inhibit mammalian complex II [34] (Table 2). We therefore evaluated whether siccanin inhibits mammalian complex I and III activities (NADH-cytochrome *c* reductase). Notably, even at concentrations as high as 500 µM, siccanin did not inhibit the mammalian complex I and III (not shown), demonstrating that siccanin is a selective inhibitor of complexes II and III from *P. falciparum*, with maximum selectivities of 57,400-fold and of more than 60-fold, respectively (Table 1). Although siccanin did not inhibit mammalian complexes I, II, or III, the growth of DLD-1 cells and HDF cells were inhibited by siccanin, which exhibited 50% effective concentrations (EC_50_s) of 34.2 ± 2.73 µM and 16.1 ± 1.21 µM, respectively (selectivities of 1.9 and 4.1-fold, Table 2). These results indicated that, in human cells, siccanin might have target(s) other than ETC enzymes.

### 2.5. Interaction of Atovaquone with Siccanin against P. falciparum In Vitro

Compounds inhibiting complex III can bind either at Q_o_ (quinol binding site facing the outer membrane) or Q_i_ (quinone binding site facing the matrix side) sites [48,49,50]. Because the growth inhibition of blood-stage parasites by siccanin was likely due to the inhibition of complex III, we tested by modified isobologram [51] based on the parasite’s LDH assay as previously described [52], whether the binding site of siccanin overlaps with that of atovaquone, which is a well-known Q_o_ site inhibitor. A pair of fractional IC_50_s for each combination of siccanin and atovaquone was plotted for the isobologram analysis. The fractional IC_50_ of siccanin was calculated by dividing the IC_50_ of siccanin combined with each atovaquone by the IC_50_ obtained for siccanin alone and plotted on the *X*-axis. Similarly, the corresponding atovaquone fractional IC_50_s were calculated and plotted on the *Y*-axis. In general, if two compounds bind at the same site, the isobologram will show an antagonistic pattern with a combination index exceeding 1. On the other hand, if the binding sites are distinct, the isobologram will show a synergistic or additive pattern, with a combination index of less than or equal to 1, respectively [53,54,55,56]. In the case of siccanin, the determined combination index was 1.0, indicating that siccanin has an additive effect when parasites are treated with the combination of siccanin and atovaquone (Figure 5c), thus, suggesting Q_i_ site as the binding site of siccanin.

### 2.6. Effect of Siccanin on P. falciparum yDHODH-3D7 and Dd2 Drug-Resistant-Panel Strains

**Figure 6:**
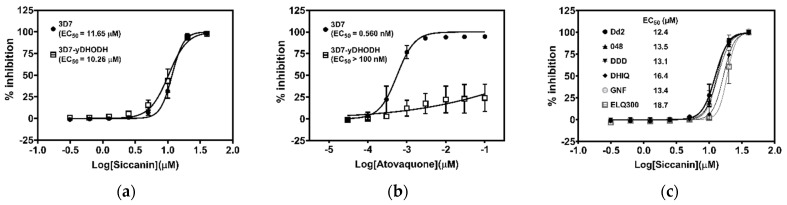
*P. falciparum* 3D7 (wild-type parent) and 3D7-yDHODH, as well as Dd2 and Dd2-derived mutant strains, are not resistant to siccanin. Growth inhibition of *P. falciparum* 3D7 and 3D7-yDHODH strains by (**a**) siccanin and (**b**) atovaquone. Data represent the means of three biological replicates, each tested in triplicate. (**c**) Growth inhibition of *P. falciparum* Dd2 (parent) and Dd2-derived mutant strains resistant to MMV390048 (048), DDD107498 (DDD), cipargamin (DHIQ), GNF156 (GNF), and ELQ300 (ELQ300). The calculated 50% effective concentration (EC_50_) values are shown next to the corresponding symbols in each panel. Data represent the means of two biological replicates, each tested in triplicate.

Previously, it was demonstrated that *P. falciparum* expressing the yeast DHODH (yDHODH) can oxidize DHO in a ubiquinone-independent manner, resulting in resistance to DHODH and complex III Q_o_/Q_i_ site inhibitors [11]. Because siccanin inhibited complex III of this parasite (Table 2), we tested whether the *P. falciparum* 3D7-yDHODH strain was resistant to siccanin. Surprisingly, siccanin was equally active against both the parent (3D7) and 3D7-yDHODH strains, showing EC_50_s of 11.65 μM and 10.26 μM, respectively (Figure 6a). In contrast, atovaquone was a potent growth inhibitor of 3D7 (EC_50_ = 0.560 nM) but not of 3D7-yDHODH (EC_50_ > 100 nM) (Figure 6b).

Next, siccanin was tested against multidrug-resistant Dd2 and drug-resistant panel strains, Dd2_048 (PI4K-S743T mutation, MMV390048^R^) [57], Dd2_DDD (eEF2-Y186N mutation, DDD107498^R^) [58], Dd2_DHIQ (ATP4-G358S mutation; cipargamin^R^) [59], Dd2_GNF (Carl-I1139K mutation; GNF156^R^) [60], and Dd2_ELQ300 (cyt *b*-I22L mutation at Q_i_ site; ELQ300^R^) [61] to investigate the cross-resistance and potential target protein(s). No siccanin resistance, however, was detected in any of these strains, for which siccanin exhibited EC_50_s for growth inhibition ranging from 12.4 μM to 18.7 μM (Figure 6c), indicating that siccanin targets other protein(s) except for the mutated gene products of these strains.

## 3. Discussion

In this study, we demonstrated that siccanin is a nanomolar-order inhibitor of complex II from an apicomplexan parasite. We also demonstrated that, while siccanin does not affect other ETC dehydrogenases, the compound shows (in addition to its activity against complex II) micromolar-order inhibition of complex III. Thus, siccanin represents a novel scaffold compared to all known complex-III inhibitors.

In mammals, mitochondrial ETC is essential for the maintenance of several processes, including energy production [62] and de novo biosynthesis of pyrimidine [63]. In contrast, the blood stage of *P. falciparum* does not depend on ETC for energy production, but instead on cytoplasmic glycolysis [64,65]. In blood-stage *P. falciparum*, ETC is essential for the de novo biosynthesis of pyrimidine, a process that is linked at the level of DHODH, Q-pool, and complex III [11] (Figure 1). Since the *P. falciparum* genome apparently does not encode a pyrimidine salvage pathway, the pathway for the de novo biosynthesis of pyrimidines and/or complex III are attractive targets for development of new antimalarial drugs [11]. Other *P. falciparum* ETC dehydrogenases, such as G3PDH and MQO, seem to be functional in the blood stage [66]. Notably, the MQO activity was higher than DHODH activity in our assay (Figure 3a). Recent studies showing that the MQO-encoding locus cannot be genetically ablated in *P. falciparum* suggests that MQO is essential for survival in the blood stage [47]. The essentiality of MQO was attributed to the key role played by MQO in linking the ETC to the fumarate cycle, given that the latter is essential for the purine salvage pathway [10]. However, other reports have suggested that PfMQO is not an appealing target [67]. Clearly, further studies will be required to determine the druggability of PfMQO. 

The knockout of the *sdha* gene in *P. falciparum* has been shown to exhibit a growth defect that is rescued by the supplementation of the culture medium with succinate, though not with fumarate [9]. In *P. berghei*, mutant parasites devoid of complex II as well as NDH2 activities have been reported to be impaired for development in the insect stage [8,68]. Complex IIs from apicomplexan parasites are known to be insensitive to classical inhibitors, and, to date, no potent inhibitors of those enzymes have been reported [27,30]. Interestingly, as shown in the present study, siccanin is a potent inhibitor of the *P. falciparum* complex II, exhibiting a biphasic inhibition pattern against this enzyme (Figure 2b). A similar biphasic inhibition pattern has been reported for *S. cerevisiae* complex II and its dinitrophenol derivative inhibitor, suggesting the existence of two quinone-binding sites [69]. The existence of two binding sites for quinone species also has been described for the quinol-fumarate reductases from *E. coli* and *Wolinella succinogenes* based on several approaches [70,71,72,73], including crystallographic studies [74]. This inference for the *P. falciparum* complex II is supported by previous reports demonstrating that malonate-sensitive NADH-fumarate reductase activity is detectable in the mitochondria-rich fraction of *P. falciparum* and also *E. tenella* (malonate is a general complex II inhibitor binding at the Fp subunit) [22,27,75]. Although similar studies of the *P. falciparum* complex II will be needed to definitively confirm this hypothesis, it is tempting to speculate that two quinone-binding sites also may exist in the plasmodial complex II; presumably, one of the quinone-binding sites has a higher affinity for siccanin than the other, resulting in the biphasic inhibition observed in Figure 2b. The first phase would be inhibited by siccanin at nanomolar-order concentration (0.016 μM), while the second phase would be inhibited by the same compound at micromolar-order concentration (8.93 μM). Interestingly, in addition to its activity against complex II, siccanin also inhibits complex III with an IC_50_ of 8.39 μM. Moreover, siccanin inhibits the *P. falciparum* complex II with over 520-fold (first phase) and 0.93-fold (second phase) greater selectively than the inhibition of complex III but with no apparent effect against mammalian respiratory chain complexes (Table 2). These results suggest that the growth inhibition of blood-stage *P. falciparum* by siccanin may be the consequence of ETC inhibition, exerted primarily at the level of complex III (rather than by the direct inhibition of the TCA cycle via complex II). However, similar sensitivity between the 3D7 and 3D7-yDHODH, as well as Dd2 and Dd2-ELQ300 towards siccanin seems to exclude complex III as its primary target in *P. falciparum*.

## 4. Materials and Methods

### 4.1. Chemicals

Siccanin (1S,4R,12S,15S,20R)-8,16,16-trimethyl-3,11-dioxapentacyclo [10.7.1.01,15.04,20.05,10] icosa-5,7,9-trien-6-ol) was obtained from Daiichi-Sankyo Pharma (Tokyo, Japan) (Figure 2a). Atovaquone was purchased from US Pharmacopeia and ICN Biomedical, Inc. (Costa Mesa, CA, USA). Unless otherwise stated, all other chemicals were purchased from Sigma-Aldrich (St. Louis, MO, USA).

### 4.2. Malaria Parasite Strains and Cultivation

The *P. falciparum* 3D7 strain was cultured, as described previously, in 3% hematocrit type A human red blood cells (RBCs) in RPMI1640 medium (Invitrogen; San Diego, CA, USA) supplemented with 25 mM sodium hydrogen biocarbonate, 10 μg/mL hypoxanthine, 40 μg/mL gentamicin sulfate, and 0.5% (*w*/*v*) Albumax II (Invitrogen) (“complete medium”) [9,52]. Cultures were maintained in a MG-70M multi-gas incubator (Taitec) under the conditions of 5% O_2_, 5% CO_2_, and 90% N_2_ at 37 °C. The medium was replaced daily [76]. Parasitemia was measured by thin blood smears stained with Giemsa. In our previous study, we generated mutant *P. falciparum* strains expressing either disrupted or full-length versions of the flavoprotein (Fp) subunit (encoded by the *Pfsdha* gene) [9]. Both strains were cultured in complete medium at 3% hematocrit supplemented with 5 nM WR99210 (Jacobus Pharmaceuticals; Plainsboro, NJ, USA) [9]. The experiments using human RBCs were performed under the guidelines of Research Ethics Committee of the Faculty of Medicine of the University of Tokyo and Institutional Review Board (IRB) of Nagasaki University (Permission nos. 10050 and no. 19, respectively). Human RBCs were obtained from The Japanese Red Cross Society. The synchronization of parasite cultures was performed by treating with 5% (*w*/*v*) sorbitol for 10 min [77].

*P. falciparum* 3D7-yDHODH was prepared and maintained as described previously [11,78,79]. *P. falciparum* Dd2, and Dd2-derived mutant strains were kindly provided by David A. Fidock [80], and maintained at Nagasaki University essentially as described in reference, except without supplementation with WR99210.

### 4.3. Assessment of Parasite Survival

Synchronized ring-form parasites (2% parasitemia) were prepared in complete medium containing 50 µM siccanin (at a fixed final DMSO concentration of 0.1% (*v*/*v*)) and distributed to 24-well plates at 1 mL/well. After incubation for 16, 24, 32, and 48 h, parasitemia was assessed under the microscope using thin blood smears stained with Giemsa.

### 4.4. Drug sensitivity Assay

Synchronized ring-form parasites (0.3% parasitemia) were prepared in complete medium containing different concentration of various compounds (at a fixed final DMSO concentration of 0.1% (*v*/*v*)) and distributed to 96-well plates at 100 μL/well. After 72 h of incubation, parasite growth was monitored by the previously described lactate dehydrogenase (PfLDH)-based assay method [81]; plates were read using a SpectraMax^®^ Paradigm spectrophotometer (Molecular Devices, Inc.; San Jose, CA, USA). EC_50_ values were calculated using Prism^®^ ver.6.01 (GraphPad; San Diego, CA, USA). Values are presented as the means of three independent experiments. 

### 4.5. Mammalian Cell Cytotoxicity Assay

Human colorectal adenocarcinoma cells (DLD-1) and Human Dermal Fibroblasts (HDF) cells were obtained from Taiho Pharmaceutical Company (Tokyo, Japan). DLD-1 cells were cultured in RPMI 1640 medium supplemented with 10% (*v*/*v*) heat-inactivated fetal bovine serum (FBS) (Gibco; San Diego, CA, USA); HDF cells were cultured in DMEM/F12 medium supplemented with 10% (*v*/*v*) heat-inactivated FBS. For both lines, cells were grown under 5% CO_2_ at 37 °C in an ACI-165D CO_2_ incubator (ASTEC; Fukuoka, Japan).

DLD-1 and HDF cells, (2.5 × 10^4^ cells/mL) in 96-well plates, were cultured for 24 h. The cells then were washed with phosphate-buffered saline (PBS) and the medium was replaced with fresh medium supplemented with 10% (*v*/*v*) FBS. Medium containing siccanin at various concentrations was added to the wells and the cells were cultured for another 48 h. Controls were treated with medium containing 1% (*v*/*v*) DMSO and 10% (*v*/*v*) FBS. Next, the cells were washed with PBS and the medium was replaced with fresh medium supplemented with 10% (*v*/*v*) FBS. An aliquot (10 µL) of CCK8 solution (Cell Counting Kit-8; Dojindo Laboratories; Kumamoto, Japan) was added to each well and the plates were incubated for another 2 h, at which point absorbance at 450 nm was monitored using a SpectraMax^®^ Paradigm spectrophotometer. The EC_50_ values were calculated using Prism^®^ ver.6.01. The selectivity was calculated as the ratio between DLD-1 or HDF versus *P. falciparum* IC_50_s. 

### 4.6. Preparation of Crude P. falciparum Mitochondrial Fraction

A synchronized parasite culture was grown for 64 h and a crude mitochondrial fraction was prepared from trophozoite-stage cells (as confirmed by Giemsa staining). Infected red blood cells were collected by centrifugation at 800× *g* for 5 min at 4 °C and incubated for 5 min at room temperature with 40 mL of 0.075% (*w*/*v*) saponin in AIM buffer (120 mM KCl, 20 mM NaCl, 10 mM PIPES (1,4-piperazinediethanesulfonic acid) buffer [pH 6.7], 1 mM MgCl_2_, 5 mM glucose). Released parasites were collected by centrifugation at 2780× *g* for 7 min at 4 °C and washed three times with AIM buffer. *P. falciparum* was suspended in MSE buffer [225 mM mannitol, 75 mM sucrose, 0.1 mM ethylenediaminetetraacetic acid (EDTA), 3 mM Tris-HCl buffer, pH 7.4] containing 1 mM phenylmethylsulphonyl fluoride, and disrupted by nitrogen cavitation at 1200 psi using a 4639 Cell Disruption Bomb (Parr Instrument Company; Moline, IL, USA). The unbroken cells and cell debris were removed by centrifugation at 800× *g* for 5 min at 4 °C. The supernatant was centrifuged at 5000× *g* for 20 min at 4 °C. The resulting pellet was suspended in 200–400 μL MSE buffer and used as crude mitochondrial fraction for all enzymatic assays, as described previously [22].

### 4.7. Measurement of P. falciparum ETC Enzyme Activities

Complex II, DHODH, MQO, NDH2, G3PDH, and dihydroorotate-cytochrome *c* (DHO-cyt *c*) reductase activities were measured, as described previously, using a V-660 spectrophotometer (JASCO; Tokyo, Japan) [14,27]. Briefly, complex II activity was measured at 25 °C in a 1 mL reaction mixture containing 45 μM 2,6-dichlorophenolidophenol (DCIP), 100 μM ubiquinone-2 (UQ2), and 2 mM KCN in 50 mM potassium phosphate buffer, pH 8.0. DHODH, MQO, and G3PDH activities were measured at 25 °C in 1 mL of the reaction mixture containing 45 μM DCIP, 100 μM UQ2, and 2 mM KCN in 30 mM Tris-HCl buffer, pH 8.0. Complex II, DHODH, MQO, and G3PDH activities were measured by monitoring the change in absorbance at 600 nm (reflecting DCIP reduction) after the reaction was initiated by adding 10 mM succinate, 500 μM dihydroorotate, 10 mM malate, or 500 μM glycerol-3-phosphate, respectively. NDH2 activity was measured at 25 °C in 1 mL of the reaction mixture containing 100 µM UQ1 and 2 mM KCN in 50 mM potassium phosphate buffer, pH 8.0. NDH2 activity was determined by monitoring the change in absorbance at 340 nm (reflecting NADH reduction) after the reaction was initiated by the addition of 20 μM NADH.

DHO-cyt *c* activity was measured at 25 °C in 1 mL of the reaction mixture containing 20 μM cytochrome *c* and 2 mM KCN in 30 mM Tris-HCl buffer, pH 8.0. The reduction of cytochrome *c* was monitored at 550 nm after initiation of the reaction by addition of 500 μM dihydroorotate [14,27]. Various concentrations of siccanin (formulated in DMSO) were added to the reaction mixtures before the initiation of the reactions. In all assays, the final concentration of DMSO was fixed at 0.1% (*v*/*v*). IC_50_ values were calculated using Prism^®^ ver.6.01.

### 4.8. Measurement of NADH-Cytochrome c Reductase Activity in Porcine Mitochondria

Porcine mitochondria were prepared as described previously [82]. NADH-cytochrome *c* (NADH-cyt *c*) activity was measured at 25 °C in 1 mL of the reaction mixture containing 1 mM MgCl_2_, 2 mM KCN, and 20 μM cytochrome *c* in 30 mM potassium phosphate buffer, pH 7.5. Activity was measured by recording the absorbance change of cytochrome *c* at 550 nm after the reaction was initiated by the addition of 20 μM NADH. Various concentrations of siccanin dissolved in DMSO were added to reaction mixtures 5 min before the initiation of the reactions. Selectivity was calculated as the ratio of mammalian to *P. falciparum* IC_50_s.

### 4.9. Isobologram Analysis with Atovaquone and Siccanin

The effect of the combination of atovaquone and siccanin was evaluated using isobologram analysis to evaluate the synergy, additivity, or antagonism between the two compounds [51,83]. Synchronized ring-form parasites (0.3% parasitemia) were prepared in complete medium containing different concentrations of siccanin and atovaquone (at a fixed final DMSO concentration of 0.2% (*v*/*v*)) and distributed to 96-well plates at 100 μL/well. After 72 h of incubation, parasite growth was monitored by the previously described PfLDH-based assay using a SpectraMax^®^ Paradigm spectrophotometer. IC_50_ values were calculated using Prism^®^ ver.6.01. Data represent the means of three independent experiments, each performed in triplicate. The isobologram analysis was performed as described previously [51,83]. For each combination assay, IC_50_s were calculated from each culture of *P. falciparum* grown in the presence of atovaquone or siccanin alone and in combination of various concentrations of the two compounds. The fractional inhibitory concentration (FIC) value was determined using the following equation:

$$\mathrm{FIC}\;=\frac{{\mathrm{IC}}_{50}\;\mathrm{of}\;\mathrm{compound}\;\mathrm{in}\;\mathrm{mixture}}{{\mathrm{IC}}_{50}\;\mathrm{of}\;\mathrm{compound}\;\mathrm{alone}}$$

The combination index (CI) value was determined using the following equation:

$$\mathrm{CI}\;=\frac{{\mathrm{IC}}_{50}\;\mathrm{of}\;\mathrm{atovaquone}\;\mathrm{in}\;\mathrm{mixture}}{{\mathrm{IC}}_{50}\;\mathrm{of}\;\mathrm{atovaquone}\;\mathrm{alone}}+\frac{{\mathrm{IC}}_{50}\;\mathrm{of}\;\mathrm{siccanin}\;\mathrm{in}\;\mathrm{mixture}}{{\mathrm{IC}}_{50}\;\mathrm{of}\;\mathrm{siccanin}\;\mathrm{alone}}$$

Values of CI < 1 represent synergism, CI = 1 represent additivity, and CI > 1 represent antagonism.

## 5. Conclusions

In conclusion, we identified siccanin as a promising antimalarial drug candidate effective not only against blood-stage parasites but also with the potential to be active against the insect stage. Such a dual-stage-targeting drug might show utility for blocking transmission as well as for the treatment of malaria patients. Notably, siccanin has no effect on mammalian respiratory chain enzymes (complex I, II, and III). However, given that siccanin inhibits the growth of DLD-1 (cancer cell line) and HDF (a normal cell line), it will be critical to determine the mammalian target of siccanin in order to increase the compound’s selectivity against the parasite for potential future development as anti-parasitic drug. 

## Data Availability

Data is contained within the article.
